# Mobile phone-based lifestyle support for families with young children in primary health care (MINISTOP 2.0): Exploring behavioral change determinants for implementation using the COM-B model

**DOI:** 10.3389/frhs.2022.951879

**Published:** 2022-11-01

**Authors:** Kristin Thomas, Margit Neher, Christina Alexandrou, Ulrika Müssener, Hanna Henriksson, Marie Löf

**Affiliations:** ^1^Division of Society and Health, Department of Health, Medicine and Caring Sciences, Linköping University, Linköping, Sweden; ^2^Department of Rehabilitation, School of Health and Welfare, Jönköping University, Jönköping, Sweden; ^3^Department of Biosciences and Nutrition, Karolinska Institute, Stockholm, Sweden

**Keywords:** implementation science (MeSH), implementation theory and research, primary healthcare, qualitative research, mobile Health (mHealth)

## Abstract

**Background:**

Obesity in childhood is a public health concern worldwide and mobile phone-based interventions (mHealth) has shown to facilitate obesity prevention. However, more research is needed on the implementation of digital tools in routine primary care. This study explored behavior change determinants for implementing a health promotion mHealth intervention (MINISTOP 2.0 app) targeting parents of 4-year-olds.

**Methods:**

Secondary data from telephone interviews (*n* = 15) with child health care nurses working within primary child healthcare in Sweden was analyzed using directed content analysis and the COM-B model.

**Results:**

Barriers for implementation included: limited knowledge about using technology and reservations about how and to what extent parents would use mHealth. Potential facilitators included nurses' openness to learn and try new tools, confidence in their role and engagement in reaching parents as well as beliefs that the app could improve practice by prompting dialogue and being a shared platform. Nurses expressed a strong professional identity and shared understanding of their practice, mechanisms that could potentially inhibit or facilitate implementation.

**Conclusions:**

Findings suggest cautious optimism regarding implementing mobile phone-based tools in child primary healthcare in terms of capability, opportunity and motivation among stakeholders. Implementation strategies such as educational outreach visits and making the intervention testable among stakeholders could further facilitate implementation in this clinical context. However, more research is needed on behavior change determinants in different stages of real-world implementation.

## Introduction

Obesity in childhood is a public health concern worldwide. According to recent figures, around 38 million children under the age of five are overweight or obese (1, 2). Sweden also shows relatively high prevalence with ~10–15% of 4-year-olds being classified as having overweight and obesity (3). Child primary health care is a key setting for obesity prevention through its reach among diverse populations and regular health visits throughout childhood from birth to school-age (4, 5). However, there are studies showing the complexity in implementing obesity prevention in routine child health care, primarily due to difficulties in getting parents on board (6–8), but also due to limited resources in health care organizations (9). Mobile phone-based interventions (so called mHealth) could facilitate obesity prevention in routine care through for example mobile applications aiming to promote healthy lifestyles among children and their families. mHealth in the area of health promotion have shown promising results on body weight and body mass index (10) as well as physical activity, smoking cessation and eating habits (10–15) and quality of care (16).

Although mHealth interventions could facilitate obesity prevention, it is unclear how these relatively novel tools can be implemented in daily routines and which determinants influence the adoption of mHealth tools in healthcare long-term (17). A systematic review showed that the most common barriers to implementing digital tools in routine healthcare were poor compatibility between the new tool and current workflow, unclear evidence of the technology, and poor organizational readiness to implement digital tools (18). A recent review on why health care professionals implement mHealth tools noted both technical aspects as well as social and organizational factors, such as the importance of ease of use, trustworthiness of the content and technical support, leadership support, peer influence and costs (17). However, the review had its focus on the implementation of mHealth targeting health services and care professionals, rather than patients per se.

Implementing mHealth in routine care can be understood as clinical behavior change e.g., child health care nurses recommend or use a mobile application during health visits. The Theoretical Domains Framework (TDF) and the COM-B model (19, 20) has been widely used in research to understand determinants of implementation in terms of behavior change. For example, the COM-B model (20) has been used to explore determinants of health behavior change among patient populations (21, 22) as well as to investigate barriers and facilitators for evidence based practice in healthcare (23). The TDF synthesize theories and constructs from 33 behavior change theories into 14 domains, argued to generate behavior. The COM-B model further consolidates these 14 domains into three overarching domains representing aspects that are argued to have to be present for a behavior to take place: “capability,” that is, an individual's capacity and competency to engage in a behavior; “opportunity,” which includes environmental factors that influence behaviors such as social support; and “motivation,” which refers to the willingness to engage in a behavior including both conscious and subconscious processes (Table 1 for a complete list of COM-B domains and TDF constructs). In summary, although mHealth can be promising tools to facilitate obesity prevention and promote healthy behaviors among families, more knowledge is needed on how mHealth can be incorporated in routine practice. Investigating behavioral determinants among nurses for using mHealth is a critical first step in understanding mHealth implementation of family-facing mHealth technology.

**Table 1:** Key findings on the inductively generated themes within each domain (capability, opportunity and motivation domains) (20), describing the role that each theoretical domain plays both regarding current health promotion work in primary child healthcare practice and implementing mHealth.

**Capability**	
Psychological capability	*Knowledge* • Knowledge related to research evidence e.g., nutrition and health • Practice-based knowledge e.g., national guidelines • Tacit knowledge e.g., understanding of target group needs • Expressed need for training on specific mHealth tools before use in practice *Cognitive skills* • Competencies used when meeting patients • Communication skills e.g., using non-dramatic terminology • Inter-personal skills e.g., validating parents to create a safe space • Perceived opportunities in using mHealth tools to facilitate communication and access health data
Physical capabilities	Not apparent in data
**Opportunity**	
Social opportunity	*Social influences* • Social norms, social support and professional identity were highly prevalent • Shared understanding among nurses regarding practice routines • Shared understanding among nurses regarding using a biopsychosocial approach
Physical opportunity	*Factors in the environmental context and resources* • “Eco-system of support” around at-risk children e.g., dieticians, physicians and teachers • Expressed need for increased collaboration among stakeholders and access to specialized care • Perceived opportunities of mHealth tools e.g., reach diverse populations through tailored mHealth resources
**Motivation**	
Reflective motivation	*Social and professional roles* • Professional identity and responsibility to support and guide parents. • Roles and responsibilities of health care professionals and target group (parents) • Perceived need for mHealth tools targeting whole families including parents and children *Beliefs about capabilities* • Confidence to engage in health promotion work and use mHealth tools • Perceived need for induction to use specific mHealth tools *Optimism* • Confidence that health care professionals and families are able to use mHealth tools
	• Perceived opportunities of mHealth tools to offer a shared platform with families, accessing material in several languages and opportunities for continuous support, monitoring and follow-up of the children and their families. • Perceived misgivings about using mHealth e.g., ensuring long term use among families *Emotions* • Both negative and positive emotions regarding using mHealth tools in practice e.g., frustration and curiosity • Expressed emotional engagement among nurses in families' efforts to adopt healthy routines

The mHealth intervention “MINISTOP” is a mobile application that was initially developed targeting parents of 4-year-olds. The app ultimately aimed to reduce the prevalence of overweight and obesity by giving support to improve diet and physical activity (MINISTOP 1.0 app). The app showed promising results on dietary and activity behaviors in a randomized controlled trial (OR: 2.0; 95% CI 1.2–3.1; *p* = 0.008) (24, 25). MINISTOP 1.0 has thereafter been refined and modified to be used within Swedish primary child healthcare targeting parents of 2–3-year-olds [MINISTOP 2.0 app (26)].

### Aim

To explore behavior change determinants for implementing a mHealth intervention (MINISTOP 2.0 app) for family nursing practice in primary healthcare.

## Methods

### Study design and setting

This study is a qualitative interview study with registered nurses working in child primary health care Secondary data (27, 28) from semi-structured interviews was analyzed using directed content analysis (29) and the COM-B model (20). The study is part of a larger research project aiming to develop and evaluate the effect of a mHealth intervention on health behavior change among parents of children aged 2–3 years [MINISTOP 2.0 trial (26)]. As part of the development of the app, interviews with nurses and parents were conducted and secondary data from interviews with nurses were used in this study. The app provides a 6-month behavior change program and includes the following features: information and practical tips provided in 13 themes with one theme released every 2 weeks (e.g., healthy snacks, fruits and vegetables, physical activity and screen time, food as rewards), a registration feature where parents can report their child's intake of fruits and vegetables, sweet drinks, physical activity and screen time and a library of healthy recipes and tips for physical activity indoor and outdoor. The parents also receive feedback in graphs and messages once a week based on their registrations. The app has also been translated and adapted for Somali- and Arabic speaking families including a large series of audio files in Somali and Arabic. As it is a web-based app, it also has a user-interface where the nurses can register new users (parents) and through that interface they are also able to follow the parental dietary and physical activity registrations mentioned above. The research was performed according to the Consolidated criteria for Reporting Qualitative Research (COREQ) checklist (30) (see Supplementary material 1).

Swedish primary child healthcare is commissioned to work with health promotion and disease prevention in a structured way. As part of this work, routine health visits follow a national health program including regular consultations with a registered nurse. In addition to height, weight, cognitive and social development of the child, these regular visits are used as a platform for the promotion of health behaviors such as physical activity and a healthy diet.

### Participants and procedure

In the original study, informants were recruited using convenience sampling with inclusion criteria: (1) currently employed at one of the participating centers and (2) willing to participate. An invite to take part in a telephone interview was sent out *via* e-mail by CA (co-author) during September 2019, and nurses registered their interest by replying to the e-mail. Invitation letters were sent to nurses (*n* = 35) at health care centers that had agreed to participate in the MINISTOP 2.0 trial (26). Recruitment was conducted from 24 primary care centers. A total of 15 nurses registered an interest to take part in interviews and were interviewed. The invitation letter consisted of information on the study including study aims, that participation was voluntary and that they could leave the study at any time.

### Data collection

Secondary data (27, 28) from interviews with child health care nurses conducted within the research project described above was used. The aim of the original study was to explore nurses' perceptions of parents' needs and concerns regarding diet and physical activity and nurses' perceptions about how the MINISTOP 1.0 app could be refined to meet the needs of the target group. In the original study, data was collected through semi-structured telephone interviews using an interview guide (Supplementary material 2) developed by the research group that has expertise in nutrition, physical activity, behavior change, and qualitative methodology. The interview guide was generated to explore nurses' perceptions about the need of the target group and preferences for using a digital tool in routines. For example, the interview guide explored nurses' perceptions regarding current work routines, needs and concerns among target group and perceptions of the MINISTOP 1.0 app. Thus, the secondary analysis used an implementation perspective, which was not done in the original study. CA (female and PhD student) conducted all the interviews which lasted on average 1 h (range 37–90 min). Field notes were taken after interviews. Informed verbal consent was obtained and recorded at the beginning of each interview. Informants were told that the interviewer was a PhD student however no relationship was established between participants and the interviewer prior to study commencement. Only participants and interviewer were present during interviews.

### Data analysis

Secondary data analysis was carried out using raw data from previously collected material. Directed content analyses (29) and the COM-B model (20) was used in data analysis. MN and KT conducted all the secondary data analysis including generating the codebook. A codebook based on the COM-B domains were used in data analysis (Supplementary material 3). Initially, key parts of COM-B were translated from English to Swedish i.e., domains (capability, opportunity, motivation and behavior). In Michie et al. (31), an extensive explanation is given of the connection between the TDF and COM-B [(31), p 87–93]. The authors point out that to identify what needs to change or when a more detailed understanding of the behavior is required, the TDF can be used to expand on COM-B domains identified in the behavioral analysis. To gain a richer understanding for the domains and how they could be operationalised for this particular dataset, constructs from the TDF were therefore also used when generating the codebook (31) (e.g., cognitive skills, beliefs about capabilities social influence etc.). Then, KT and MN individually generated codebooks based on the translated domains and constructs. The two codebook drafts were discussed and consensus about one final codebook was reached (see Supplementary material 3).

KT and MN performed the secondary data analysis separately using the codebook to ensure consistency. However, inter-coder reliability was strived for through regular meetings between KT and MN throughout the analysis process. Firstly, all the transcripts were read through to gain an understanding and impression of the data as a whole. Then, all data was reviewed for content and coded according to the pre-defined categories in the codebook. This coding phase involved identifying data that corresponded with, or exemplified, COM-B domains by using the codebook. Only data that corresponded to pre-defined categories were coded. Preliminary findings (sorting and coding of data) were discussed between MN and KT in an iterative process until agreement was reached. In a final step, KT and MN together drafted text that described each category including selecting citations that could illustrate the content. KT drafted the first version of the results section for this manuscript.

## Results

The study aimed to explore determinants for implementing a parent-oriented mHealth intervention in health promotion practice in primary child healthcare. In total, 15 nurses from nine primary child healthcare centers took part in telephone interviews. The participants were on average 47 years of age (between 37 and 55) and had on average worked in their profession for 7 years (between three and 11 years). Implementation referred to nurses introducing the MINISTOP 2.0 app to parents of 4-year-olds within family nursing practice during routine health visits. The analysis explored how nurses perceived their current health promotion practice and used the COM-B model (20) to systematically map determinants in data. Results are presented below for each COM-B domain.

### Behavior

In the interviews, the nurses described and reflected on what their current work routines entailed. Nurses expressed health promotion work to be continuous, preventative, and comprehensive, aiming to support families from infancy to school-age. The work included monitoring children's social, psychological, and physical health mainly through meeting families during scheduled visits and referring to specialists when needed. In their conversations with parents, they provided information about health risks related to overweight and obesity in childhood and guided parents through healthier living. This is the professional work in which the MINISTOP 2.0 app would be introduced. “*Within child health care all health visits are preventative … so we talk about growth curves and health behaviours in all visits at the clinic*” (Informant 7).

### Capability

In the COM-B model, capability refers to psychological and physical capability to engage in a behavior. The data mainly included nurses exhibiting knowledge and cognitive skills to do health promotion work and to use mHealth in this practice. Thus, aspects of physical capabilities were not apparent in the data.

Nurses expressed extensive knowledge on the responsibility, approach, and routines of the primary child healthcare organization. Their knowledge encompassed both research evidence about for instance nutrition and health, practice-based knowledge about e.g., national guidelines and pedagogical tools as well as tacit knowledge about parents' everyday life and concerns. Furthermore, the nurses stressed the need to keep themselves updated to ensure quality of care. Nurses kept updated through their contact with parents, learning from cultural bridge-builders, other colleagues and searching the Internet. ”*I have gained so much from them [bridge builders] …a lot…a culture competency which I didn't know…yeah didn't realise existed before I started here*” (Informant 11). In addition, nurses highlighted the importance of staying curious and open-minded about the meaning of food in different cultural contexts to be able to support parents. “*It is quite difficult to know I think … if I don't come from the same food culture as the person I meet…then I don't know exactly what they eat*” (Informant 14). Regarding using mHealth in practice, the nurses highlighted the need for training about specific tools to increase capability, including perhaps testing MINISTOP themselves before disseminating it among parents. ”[sigh] *Yeah… you need training as well of course… and… guidelines on how … yes how to use it [mobile application] and that we get a united way of working is important*” (Informant 8).

Cognitive skills referred to the competencies the nurses used when meeting parents. These skills included for instance adopting a light-hearted approach, using non-dramatic terminology, and validating parents' concerns to create a safe environment for parents to share information. Using one's cognitive skills also involved to always assess the situation and the individual in front of you and changing communication techniques accordingly.” *what experience does the parent have?//…the approach becomes who are you?…and what do you need from me?…these things I need to explore before I give advice*” (Informant 10). Thus, health promotion work was described as a two-way process with shared responsibility between professional and parent. Although nurses expressed confidence in their role, child obesity was described as a potentially sensitive subject that can provoke strong emotions among parents such as pride, obstinance, guilt and shame. ”*Others blame themselves and believe that I have actually done wrong as a mother, yeah, and so there is some shame and guilt in this*” (Informant 9). Regarding their cognitive skills, the data suggested that there was a capability among nurses to use mHealth in current health promotion work. For example, nurses expressed that mHealth could be used together with parents and could facilitate communication by making the topic of obesity less dramatic. Also, the potential of accessing data on families continuously through the mobile phone was thought to enable monitoring long-term and ultimately improve the communication during visits.

### Opportunity

Within COM-B, opportunity refers to social and physical opportunities to engage in a behavior. The data included aspects on social influences and factors in the environmental context relating to health promotion work and using mHealth in practice.

Data on social influences, conveyed that social norms, social support and group-identity were highly prevalent in nurses' health promotion work. This was illustrated by nurses' shared understanding and acceptance of practice routines and understanding of health whereby social, psychological, and physical health concerns were continuously monitored and addressed, from infancy to school-age. “*In child primary health care we work preventative at all visits…so we talk about this with children and growth curves and lifestyle…at all visits*” (Informant 7). Furthermore, nurses expressed that health promotion work is more than promoting healthy behaviors: it is also about parenthood and inducing confidence in parents to be able to follow through with health behavior change.

Regarding, environmental context and resources, nurses described a network of actors around at-risk children that could be potential resources in promoting health such as nurses, dieticians, physicians, specialists, interpreters and bridge-builders and teachers in nursery. ”*What happens at home…what happens there…because of course we call them back after six months but what happens…in the family at nursery at the grandparents?*” (Informant 7). Nurses expressed that collaboration within this “ecosystem of support” can be challenging but also rewarding for instance through working with bridge-builders to learn about different food cultures. Nurses talked about needing more resources such as increased access to specialized care as well as hands-on tools and materials that could facilitate communication and dissemination of information. For example, nurses talked about opportunities with future mHealth interventions to be available in several languages including pictures which are valid across cultures. “*And especially if there is something we use these pictures…or if you have difficulties with the language or…then it is very good to have a picture*” (Informant 7). In some cases, nurses perceived that the pedagogic and information materials that they currently had access to were not up to date and that their methods of counseling were not attractive to the families they served. “*But the fact that there is no…that there…if we talk about balanced diet examples…everything is in Swedish…everything is adapted to how a Swedish plate looks like…not how it looks for Somali families or a family from China//here we have so much to learn…so much to learn*” (Informant 13).

### Motivation

Within the COM-B model, motivation refers to both reflective (conscious) and automatic (subconscious) processes to engage in a behavior. The data mainly included motivation in terms of social and professional roles, beliefs about capabilities, optimism, intention and emotions associated with health promotion work and using mHealth in practice. Thus, other dimensions of motivation that is described in COM-B such as reinforcement, goals and beliefs about consequences were not apparent in data.

Regarding social and professional roles, nurses conveyed a strong professional identity and responsibility to support and guide parents. Nurses also described boundaries for their responsibility, or ability to help, with families that despite several efforts, were difficult to reach. “*You know [sighing] sometimes you don't get there…sometimes there is no interest…sometimes you can't do it*” (Informant 14). Nurses highlighted that obesity often is a problem in the family as a unit whereby both professionals and parents have a responsibility. Relating to this, the nurses sought for mHealth interventions that targeted whole families rather than parents per se by for instance engaging children in the material. Furthermore, the nurses expressed that recruiting parents and supporting parents' long-term use of MINISTOP were important and part of their professional role.

Data on beliefs about capabilities included professional confidence to engage in health promotion work but also about using mHealth. Nurses were confident in performing health promotion work also stating that they perceived that their parent-and -child interaction skills developed over time. When asked to give their initial reaction on implementing a mHealth in this practice, nurses expressed that they would like to try out using the tool but that there could be a need for staff introduction and training to be able to integrate the tool fully in practice. ”*What is needed is that everybody works with the app the same way…because sometimes we meet each other's children and…so it is important that everybody has the same training so we work the same way…refer similar cases*” (Informant 4).

Nurses expressed both optimism and challenges toward working with mHealth in routine practice. Apart from an optimism that both themselves and parents are technically savvy, nurses could identify additional benefits for example, the opportunity for parents to constantly access information and support as opposed to only when visiting the clinic. Nurses expressed that the mHealth intervention could facilitate the work with hard-to-reach families, especially parents of high-risk children and parents with poor reading and writing skills through alternative channels such as audio and video. ”*It is actually those families…yes…if we look in general…if we look at our families here now…so issues around diet…around teeth …around overweight then it is problem in this group…that's where it is most difficult to reach…and of course you can influence…we can see…but can you reach…can you…can we use a tool that we use together then it would be easier…I believe*” (Informant 13). Other benefits mentioned were the potential of having a shared platform with parents, accessing material in several languages and opportunities for continuous support, monitoring and follow-up of the children and their families. Nurses also expressed misgivings about using mHealth, such as the added distraction for parents,” *That…they use the app…exactly…when do I use the app…that's the point…as a parent…do I look at that instead of my child?*” (Informant 10). Other challenges described were achieving good communication in technical solutions, and difficulties in achieving long-term use among parents. ”*Then it is this with the …the in-person meeting…to like still…whatever app you have…to be able to refer to the in-person meeting*” (Informant 10). As their advice on screen activity was usually about limiting the time spent with media, they worried about introducing another screen-dependent activity in the lives of the families they served. Although they indicated that modern families were very cognizant about using mobile phones in general, they also reflected on the risk of MINISTOP “disappearing” in the crowd of mobile applications that parents use every day. In general, the nurses expressed an optimism that MINISTOP would fit with current routines and feasibility to recruit families. ”*It fits very well in our like when we talk about…diet and sleeping and screen time and activity and so on…this is exactly what we discuss at every visit*” (Informant 7).

The nurses described that the goal of monitoring health behaviors in families in their practice led them to take intentional charge of the conversations with parents in different ways. These intentions also led them to provide suggestions concerning the mHealth and the desirable functions for staff. Although they were used to using mobile phone applications in general, they were not very familiar with the MINISTOP application, but they stated that they welcomed new ways to manage their health promotion task. Nurses expressed that parents' intention to work with mHealth would depend on the characteristics of the intervention itself, but also on the health behavior interest of the family. They believed that mHealth would be useful for health promotion, but also that outside support was necessary to keep the issue at the top of the family's agenda.

The nurses expressed negative emotions like frustration, resignation and worry but also positive emotions such as excitement and curiosity regarding their work and using mHealth in practice. ”*Yes…no but there is nothing that I feel at the moment…that no but…when I see this it feels really exciting I think…we can hope that the parents also think that…or will think* [laughter]” (Informant 15). The nurses were emotionally engaged in the success and failure of their efforts to involve parents in the health promotion conversations, and disappointed when the child's health data indicated that results were lacking. As conversations about child obesity could lead to parental emotions such as shame and blame, nurses expressed that they experienced negative emotional stress in these situations. When asked to reflect on the use of MINISTOP by parents, the participants expressed feeling hesitation, but also excitement and curiosity, and they expressed that they looked forward to working with the app.

In summary, potential barriers for behavior change were limited knowledge and reservations among nurses regarding the use of the intervention among the target group. Potential facilitators for behavior change were nurses' openness, confidence and engagement in their professional role and beliefs that digital tools could improve practice.

## Discussion

This study explored behavior change determinants for implementing an mHealth intervention (MINISTOP 2.0 app) in current health promotion practice in primary child healthcare. Determinants in terms of both barriers and facilitators for implementation were identified. Limited knowledge about MINISTOP and reservations about how and to what extent parents would use the intervention were identified as the main possible hinders. Potential facilitators were nurses' openness to learn and try innovations, nurses' confidence and engagement in their professional role and beliefs that mHealth could improve practice by prompting dialogue and being a shared platform. Finally, nurses expressed a strong professional identity and shared understanding of their practice, mechanisms that could potentially hinder or facilitate implementation of mHealth.

One of the potential barriers for implementing MINISTOP 2.0 was the capability among nurses to use the intervention. The COM-B model posits that the capability and motivation to engage in a behavior are interrelated, and would together with behavioral opportunities, contribute to adoption of mHealth (20). Although nurses expressed these hinders, our data indicated that nurses also exhibited capability (in terms of cognitive skills), opportunity and motivation to use and implement the MINISTOP in their daily routines. Indeed, well-known facilitators of practice change were observed such as nurses' openness to change and beliefs that the mHealth intervention would improve practice. In contrast, previous research in pediatric care has shown that poor buy-in and engagement among adopters together with limited time and information are typical barriers to implement mHealth (31). Innovations that are compatible with existing norms, values and ways of working have shown to easier engage adopters which could partly explain our findings. Indeed, the child primary healthcare context was found to be characterized by a strong professional identity, engagement, and long-term relationship with families. All these mechanisms have the potential to facilitate implementation of any innovation that is compatible with these notions and ethos (32). The nurses expressed that MINISTOP was compatible with their work for example by taking a preventative and holistic approach to health and offering information to parents.

Research on implementation has shown that an important determinant is how end-users perceive different attributes of the intervention so called innovation characteristics. Indeed, the central tenet of the Diffusion of Innovations theory (33) is that how attributes of a technology are perceived by stakeholders will influence implementation. Facilitating attributes include perceived relative advantage (the intervention is perceived to be superior to existing routines), complexity (an intervention is simple to use and understand), compatibility (an intervention matches established routines and norms), observability (potential benefits of an intervention is visible), and trialability (an intervention can be tested prior full-scale implementation) of an innovation (33). The possible barriers can be understood as the complexity and trialability of MINISTOP 2.0, that is, nurses expressed wanting more knowledge about the intervention and testing it before full-scale implementation. Effective implementation strategies to facilitate implementation could thus be educational outreach visits and making MINISTOP testable among nurses to promote familiarity (34). However, to fully facilitate the implementation of digital interventions in routine practice, implementation strategies need to target all barriers for change. Thus, strategies need to be systematically developed based on a thorough investigation of determinants specific for this particular context and intervention (31, 35).

Our findings echo existing literature on mHealth implementation. Indeed, a systematic review on mHealth adoption in healthcare highlighted perceived usefulness and familiarity, training and access to resources to be key determinants of successful implementation (17). Usefulness in the mHealth implementation literature typically refers to that mHealth interventions will not only fit current routines, but also make valuable additions to these. Our findings can add to the understanding of the usefulness of mHealth tools in healthcare, knowledge that is central for intervention development and implementation. In our data, nurses spoke about the usefulness of MINISTOP 2.0 in terms of the opportunities to monitor behavior over time and to create a shared platform incorporating multiple stakeholders. Nurses described how obesity prevention engaged a network of actors and that mHealth hold great potential in providing a shared platform in this work. A qualitative study on the use of mHealth in general practice similarly characterized usefulness as creating shared platforms for patients and healthcare providers (36). However, it can be a challenge for future mHealth designs to, on one hand, include multiple stakeholders and, on the other hand, tailor content to specific target groups. mHealth interventions can be designed to be a platform where information is disseminated to patients, alternatively, mHealth can be designed as a dynamic tool, a place, where different stakeholders can communicate and share experiences continuously. In addition, the nurses voiced that they would like mHealth interventions to be a tool shared between themselves and parents. This could for example be done by parents registering their health behaviors, data that nurses would have access to, and used in consultations.

### Methodological considerations

A strength of the study is the use of a theory-based codebook in data analysis which supported neutrality and consistency in the analysis process (37). Trustworthiness and scientific rigor were strived for in several ways (37). Researchers with varied research backgrounds and competencies were involved during study design, data collection and analysis which could have increased credibility. In addition, investor triangulation was used in data analysis to ensure dependability of the interpretation of data. The theory-based codebook enabled systematic data analysis which together with investigator triangulation could have increased dependability and confirmability. Furthermore, we have strived to increase transferability by providing detailed description of the procedure and thick descriptions of the results, illustrated by quotes from data. Transferability may have also been increased by the fact that healthcare centers, in which informants work, were located in diverse socioeconomic and geographical areas.

A potential challenge with using secondary data is that the interview material and the original interview guide may not adequately answer the study aim. Therefore, before starting data analysis, we read through the material to assess whether it included sufficient scope and depth to capture our study aim. We deemed that the data were sufficient to investigate conditions for mHealth implementation among nurses and offered valuable insights in this regard. However, including interviews with practice managers and regional managers could have strengthened the study. The study offers the perspective of registered nurses and knowledge on implementation in the healthcare visit setting. Future studies could include other groups of informants to enrich our understanding of implementation on other levels of the primary care organization.

The study explored future, potential, determinants for implementing an innovation in current practice, rather than actual experienced determinants. Perceived determinants prior an implementation is not necessarily the same as the ones that are later experienced during actual implementation. However, the nurses were experienced in health promotion work and showed extensive knowledge about obesity prevention and preconditions increasing the validity of the findings. Future research needs to investigate readiness to change and determinants among healthcare professionals with hands-on experience with mHealth implementation. This study adopted a point of departure in current practice routines to understand determinants for future implementation.

## Conclusions

This study indicates cautious optimism regarding the preconditions for implementing mHealth in child primary healthcare in terms of capability, opportunity and motivation among stakeholders. Implementation strategies such as educational outreach visits and making the intervention testable among stakeholders could further facilitate implementation in this clinical context. However, more research is needed on the impact of behavior change determinants in different stages of real-world implementation.

## Data availability statement

The raw data supporting the conclusion of this article can be made available by the authors on reasonable request.

## Ethics statement

The studies involving human participants were reviewed and approved by the regional Ethical Review Board of Uppsala, Sweden (ref no 2019-02747; 2020-01526). The patients/participants provided their written informed consent to participate in this study.

## Author contributions

ML is the principle investigator for the MINISTOP 2.0 trial, including this qualitative study. CA and HH performed the data collection in the original study. KT and MN conducted the secondary data analysis. KT drafted the first version of the manuscript with significant contributions from ML, CA, UM, MN, and HH. All authors read and approved the final version of the manuscript.

## Funding

This research was conducted within the MINISTOP 2.0 trial and funded by several grants: Swedish Research Council for Health, Working Life and Welfare (FORTE, 2018-01410; PI ML), Region Östergötland (LIO-893101; PI ML), Region Östergötland (LIO-920441; HH), and Lions Research Fund (PI HH). None of the funders have had input on study design, data collection, data analysis, or preparation of the manuscript.

## Conflict of interest

The authors declare that the research was conducted in the absence of any commercial or financial relationships that could be construed as a potential conflict of interest.

## Publisher's note

All claims expressed in this article are solely those of the authors and do not necessarily represent those of their affiliated organizations, or those of the publisher, the editors and the reviewers. Any product that may be evaluated in this article, or claim that may be made by its manufacturer, is not guaranteed or endorsed by the publisher.
