# Supplementary material for: Mobile phone-based lifestyle support for families with young children in primary health care (MINISTOP 2.0): Exploring behavioral change determinants for implementation using the COM-B model
Source: Front Health Serv. 2022 Nov 1;2:951879. doi: 10.3389/frhs.2022.951879 (PMC10012784; doi:10.3389/frhs.2022.951879)
Supplement: Supplementary file 2 [file Table_1.DOCX]

Supplementary material 2: Interview guide

| Interview questions | |
| --- | --- |
| *Interview questions aiming to capture nurses’*   - *Current working routines* - *Perceived parental challenges regarding healthy living* - *The use of a parent-oriented mHealth tool in professional work* | |
|  | What are your current health practice routines within primary child health care, in terms of the promotion of healthy diet and activity, to prevent future adverse health outcomes in children in general? |
|  | What are your current practice routines for treatment of children with overweight or obesity? |
|  | What problems or difficulties do you experience/encounter with the current way of working? |
|  | If you think in terms of obesity treatment, what problems or difficulties do you experience with the current way of working? |
|  | What do you find is most challenging for families/parents when it comes to healthy eating, physical activity and screen time? |
|  | Do you experience any differences in challenges between families/parents that have children with overweight or obesity? |
|  | What are your experiences/perceptions of reaching families in need of support in general, with information on health behaviours for children? |
|  | What are your experiences/perceptions of reaching families where the child has obesity, in terms of health behaviours? |
|  | We want to explore how to best support health behaviours in children through a smartphone app. In general, what advantages/disadvantages do you see with using such an app for prevention? |
|  | Could you see any advantages/disadvantages with using such an app for childhood obesity treatment? |
|  | Are there any benefits with using such an app for general prevention, compared to current working methods?  a) For the families?  b) For you as a health care professional?  c) If you think about it in terms of childhood obesity treatment, are there any benefits compared to current working/practice methods?  d) Are there any specific (population) groups that you believe could benefit more from extra support through an app? |
|  | If you think in terms of prevention, how do you think it would work to use such an app? |
|  | How do you think it would work to use such an app, if you think in terms of childhood obesity treatment? |
|  | If you were to recommend an app like this to families, what type of content and/or features do you think the app should include, in order for you to be willing to recommend it for prevention? |
|  | If you think in terms of obesity treatment, what would be important for the app to include in order for you to be willing to recommend it to families? |
|  | What would need to change/is there anything that needs to change in your current health practice routines, for you and your colleagues to be able to use the app, if you think both in terms of prevention and in terms of obesity treatment? |
|  | Would it be difficult for you to use the app, or could you see the app as a complement? |
| *Interview questions regarding the features and content of the MINISTOP 1.0 app. The nurse is shown screenshots of the app features and content to be able to reflect on these.* | |
|  | Overall, what do you think about the content in the app? (both in terms of prevention and obesity treatment) |
|  | Are the 12 different themes in the app relevant to the target group (parents with children aged 2-3 years)? |
|  | Are there any themes that you are missing and would like us to add? |
|  | Is there anything else missing? |
|  | What is your opinion on families receiving information about several health behaviours (i.e., diet, physical activity and screen time) at the same time in an app? |
|  | Do you use any other app in your work/practice? How does that work? |
|  | Is there anything else we should think about when developing the app further? |
|  | |
