# Supplementary material for: Mobile phone-based lifestyle support for families with young children in primary health care (MINISTOP 2.0): Exploring behavioral change determinants for implementation using the COM-B model
Source: Front Health Serv. 2022 Nov 1;2:951879. doi: 10.3389/frhs.2022.951879 (PMC10012784; doi:10.3389/frhs.2022.951879)
Supplement: Supplementary file 3 [file Table_2.DOCX]

Title: Mobile phone-based lifestyle support for families with young children in Primary Health Care (MINISTOP 2.0): Exploring behavioral change determinants for implementation using the COM-B model.
Authors: Kristin Thomas, Margit Neher, Christina Alexandrou, Ulrika Müssener, Hanna Henriksson and Marie Löf.

| Supplementary material 3. Codebook based on the COM-B model and Theoretical Domains Framework [28]. | | | |
| --- | --- | --- | --- |
| **COM-B^1^** | **TDF^2^ domain**  **Definition** | **Theoretical constructs represented in each domain** | **Codebook guidance with potential topics of data** |
| Psychological capability | Knowledge | Knowledge (including knowledge of condition/scientific rationale; procedural knowledge; knowledge of task environment | - Expressions of knowledge about working preventative and using Ministop - Expressions of knowledge about current practice routines - Expressions of knowledge about health behaviour and health risk behaviours - Expressions of knowledge about available resources and opportunities at the workplace - Implicit knowledge about current practice routines |
|  | Cognitive skills | Skills; skill development; competence; ability; interpersonal skills; practice; skill assessment | - Expressed competency in working preventative and using Ministop - Expressed competency to reach families from diverse cultural backgrounds - Expressed competency to use tools e.g., material and motivational interviewing in health visits - Expressed competency to assess need of care and build rapport with families - Expressed competency relevant for practice routines |
|  | Memory, attention and decision processes | Memory attention; attention control; decision-making; cognitive overload/tiredness | - Expressions of implementing preventative work and using Ministop |
|  | Behavioural regulation | Self-monitoring; breaking habit; action planning | - Described strategies or activities that aim to brake habits in routine practice - Awareness of different health behaviours and health risk behaviours - and their consequences - Expressed plans for behaviour or practice changes |
| Physical capability | Physical skills | Skills; skill development; competence; ability; interpersonal skills; practice; skill assessment | - Expressed capability of how to implement preventative work and using Ministop |
| Social opportunity | Social influences | Social pressure; social norms; group conformity; social comparisons; group norms; social support; power; intergroup conflict; alienation; group identify; modelling | - Perceived social support for working preventative and using Ministop - Expressions of norms for working preventative and using Ministop - Expressions of group identity that could influence working preventative and using Ministop |
| Physical opportunity | Environmental context and resources | Environmental stressors; resources/material resources; organizational culture/climate; salient events/critical incidents; person x environment interaction; barriers and facilitators | - Perceived stressors at the workplace that could influence preventative work and using Ministop - Material resources to working preventative and using Ministop - Expressions of an organizational culture that influence working preventative and using Ministop in a positive or negative way - Other factors that influence the work in a positive or negative way |
| Reflective motivation | Social/professional role and identity  A coherent set of behaviours and displayed qualities of an individual in a social or work setting | Professional identity; professional role; social identity; identity; professional boundaries; professional confidence; group identity; leadership; organizational commitment | - Perceived compatibility with professionals’ standards and identity - Expressions of professional identity relevant for working preventative and using Ministop - Expressions of professional roles relevant for working preventative and using Ministop - Boundaries between professions (in relation to e.g., roles and responsibilities) - Perceptions of the role and influence of leadership relevant to working preventative and using Ministop |
|  | Beliefs about capabilities | Self-confidence; perceived competence; self-efficacy; perceived behavioural control; beliefs; self-esteem; empowerment; professional confidence | - Expressions of confidence relevant to working preventative and using Ministop - Expressions of self-efficacy in relation to working preventative and using Ministop - Expressions of control in own work situation |
|  | Optimism | Optimism; pessimism; unrealistic optimism; identity | - Beliefs that everything will work out fine - Beliefs that goals will be reached - Expressions of pessimism - Unrealistic optimism |
|  | Intentions | Stability of intentions; stages of change model; transtheoretical model and stages of change | - Expressions of intended decisions or behaviours relevant to working preventative and using Ministop - Decisive expressions of implementing new work routines or using mobile health tools in practice |
|  | Goals | Goals (distal/proximal); goal priority; goal/target setting; goals (autonomous/controlled); action planning; implementation intention | - Expressed goals for working preventative and using Ministop - Expressed ideas for working preventative and using Ministop - Hopes relevant to what they believe will be accomplished with working preventative and using Ministop - Expressed expectations of preventative work and using Ministop |
| Automatic motivation | Reinforcement | Rewards (proximal(distal, valued/not valued, probable/improbable); incentives; punishment; consequences; reinforcement; contingencies; sanctions | - Described incentives to work preventative and using Ministop - Perceived punishments or negative consequences of not working preventative and using Ministop |
|  | Emotion | Fear; anxiety; affect; stress; depression; positive/negative affect; burn-out | - Emotional expressions in relation to working preventative and using Ministop - Expressed fear and/or worry in relation to working preventative and using Ministop - Expressions of stress in relation to working preventative and using Ministop - Expressions of positive and/or negative emotions in relation to working preventative and using Ministop |
| ^1^COM-B model [20]  ^2^Theoretical Domains Framework [19] | | | |
